# Giant All-Optical Modulation of Second-Harmonic Generation Mediated by Dark Excitons

**DOI:** 10.1021/acsphotonics.1c00466

**Published:** 2021-07-13

**Authors:** Yadong Wang, Susobhan Das, Fadil Iyikanat, Yunyun Dai, Shisheng Li, Xiangdong Guo, Xiaoxia Yang, Jinluo Cheng, Xuerong Hu, Masood Ghotbi, Fangwei Ye, Harri Lipsanen, Shiwei Wu, Tawfique Hasan, Xuetao Gan, Kaihui Liu, Dong Sun, Qing Dai, F. Javier García de Abajo, Jianlin Zhao, Zhipei Sun

**Affiliations:** † MOE Key Laboratory of Material Physics and Chemistry under Extraordinary Conditions, and Shaanxi Key Laboratory of Optical Information Technology, School of Physical Science and Technology, Northwestern Polytechnical University, Xi’an 710129, China; ‡ Department of Electronics and Nanoengineering, Aalto University, Espoo 02150, Finland; § ICFO-Institut de Ciencies Fotoniques, The Barcelona Institute of Science and Technology, 08860 Castelldefels (Barcelona), Spain; ∥ International Center for Young Scientists, National Institute for Materials Science, Tsukuba 305-0044, Japan; ⊥ CAS Key Laboratory of Nanophotonic Materials and Devices, CAS Key Laboratory of Standardization and Measurement for Nanotechnology, CAS Center for Excellence in Nanoscience, National Center for Nanoscience and Technology, Beijing 100190, China; # Changchun Institute of Optics, Fine Mechanics and Physics, Chinese Academy of Sciences, Changchun, Jilin 130033, China; ∇ International Cooperation Base of Photoelectric Technology and Functional Materials, and Institute of Photonics and Photon-Technology, Northwest University, Xi’an 710069, China; ○ Department of Physics, University of Kurdistan, Pasdaran St., Sanandaj 66177-15177, Iran; ◆ School of Physics and Astronomy, Shanghai Jiao Tong University, Shanghai 200240, China; ¶ State Key Laboratory of Surface Physics, Key Laboratory of Micro and Nano Photonic Structures (MOE), and Department of Physics, Fudan University, Shanghai 200433, China; + Cambridge Graphene Centre, University of Cambridge, Cambridge CB3 0FA, United Kingdom; ■ State Key Laboratory for Mesoscopic Physics and School of Physics, Peking University, Beijing 100871, China; △ International Center for Quantum Materials, School of Physics, Peking University, Beijing 100871, China; □ QTF Centre of Excellence, Department of Applied Physics, Aalto University, Espoo 02150, Finland; ● ICREA-Institució Catalana de Recerca i Estudis Avançats, Passeig Lluís Companys 23, 08010 Barcelona, Spain

**Keywords:** Second-Harmonic Generation, Dark Excitons, Bright Excitons, Transition Metal Dichalcogenides Monolayers, Ultrafast Optical Modulation, Optically-Modulated Excitonic Strength

## Abstract

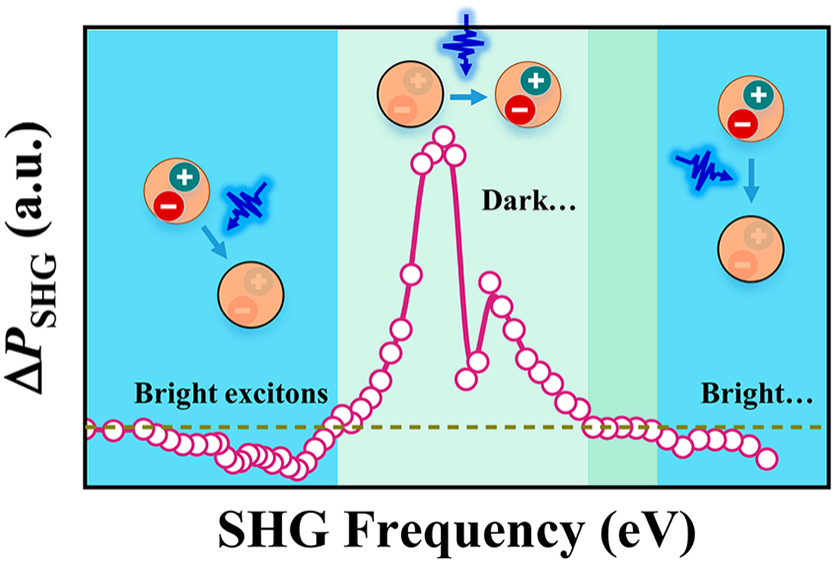

All-optical control of nonlinear photonic processes in nanomaterials is of significant interest from a fundamental viewpoint and with regard to applications ranging from ultrafast data processing to spectroscopy and quantum technology. However, these applications rely on a high degree of control over the nonlinear response, which still remains elusive. Here, we demonstrate giant and broadband all-optical ultrafast modulation of second-harmonic generation (SHG) in monolayer transition-metal dichalcogenides mediated by the modified excitonic oscillation strength produced upon optical pumping. We reveal a dominant role of dark excitons to enhance SHG by up to a factor of ∼386 at room temperature, 2 orders of magnitude larger than the current state-of-the-art all-optical modulation results. The amplitude and sign of the observed SHG modulation can be adjusted over a broad spectral range spanning a few electronvolts with ultrafast response down to the sub-picosecond scale via different carrier dynamics. Our results not only introduce an efficient method to study intriguing exciton dynamics, but also reveal a new mechanism involving dark excitons to regulate all-optical nonlinear photonics.

## Introduction

Second-harmonic generation (SHG), a nonlinear optical process originating in the second-order response of noncentrosymmetric materials, is arguably the most commonly used nonlinear optical effect.^1^ An efficient control of SHG is vital for various important applications that include optical data processing, spectroscopy, and quantum photonics. In previous works, all-optical control of SHG^2^ has been demonstrated in semiconductors^3^ as well as in metallic^4^ and hybrid structures,^5−7^ primarily relying on optically induced electric fields and hot electrons. However, the reported modulation of SHG in noncentrosymmetric materials is generally very weak (typically with enhancement factors ≲ 4). This lack of efficient all-optical modulation strategies represents a major bottleneck toward the development of emerging and future applications, such as quantum photonics and on-chip nonlinear devices.

In recent years, two-dimensional (2D) transition metal dichalcogenides (TMDs) have emerged as a powerful platform for applications in photonics and optoelectronics,^8^ including nonlinear optics.^9^ Specifically, excitons introduce strong resonances in the optical response of TMDs, which dominate their linear and nonlinear optical properties aided by the extreme quantum confinement and reduced screening in these materials.^9−12^ As a consequence, significant research efforts have been devoted to investigate and exploit the enhancement of the nonlinear optical response in TMDs,^13−21^ which is fascinating from both fundamental and applied perspectives. Interestingly, excitonic Rydberg states exhibit general characteristics of hydrogen-like atoms, possessing a series of discrete optically accessible (bright; 1*s*, 2*s*, ...) and optically forbidden (dark; 2*p*, 3*p*, ...) states, as determined by optical selection rules.^22^ Through strong resonant enhancement of bright excitons, SHG can be actively tuned using several methods, such as electrical and chemical doping.^19−21,23−27^ However, the influence of dark exciton states on nonlinear optics has remained largely unexploited.^22,28^

Here, we demonstrate giant all-optical modulation of SHG within a broad spectral range in monolayer TMDs at ultrafast speed (down to ∼500 fs). Our results confirm that SHG modulation is strongly related to dark excitonic states, with the SHG modulation being enhanced by the creation of dark excitons and suppressed by bright excitons. The measured enhancement of SHG reaches a factor as large as ∼386. By combining bright- and dark-exciton resonances, we achieve a dramatic modulation of the SHG amplitude, sign, and response time over a wide spectral range. We explain our results by performing first-principles calculations supporting the leading role of optically pumped dark excitons. Our study emphasizes time-resolved SHG spectroscopy as an efficient way to investigate high-order excitonic states and their dynamics in 2D materials and their heterostructures. Additionally, our demonstration of a giant enhancement in the nonlinear optical processes of TMD materials holds great potential for applications in all-optical devices.

## Results and Discussion

**Figure 1:**
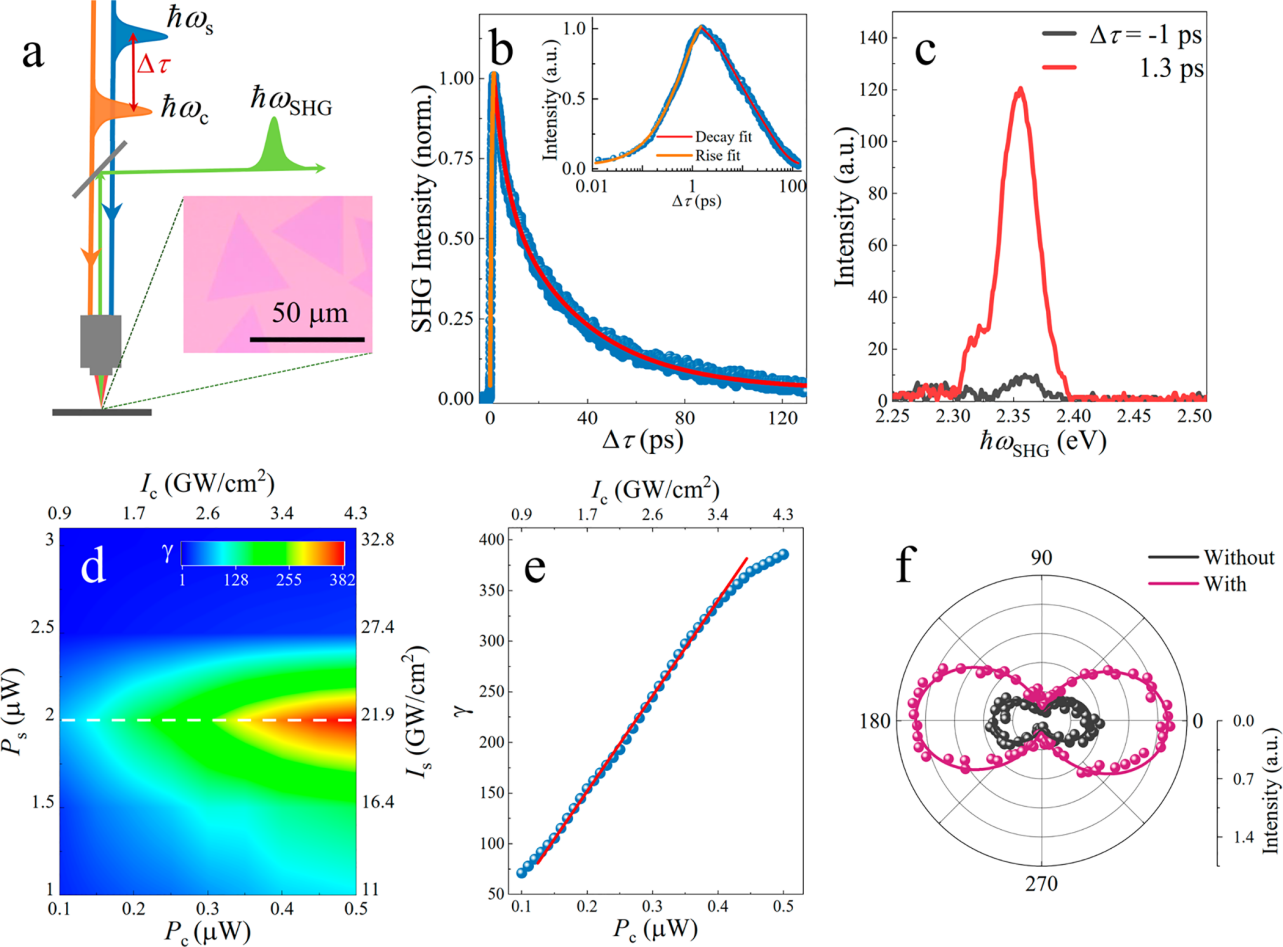
Giant SHG enhancement in MoS_2_. (a) Sketch of the experimental setup. The inset shows an optical image of the probed MoS_2_ flakes. (b) Normalized SHG intensity as a function of delay time Δτ. The average power of the control and seed light is ∼0.5 and ∼2 μW, respectively. The pulse duration is ∼230 fs. The inset shows a semilog rendering of the same data. (c) SHG spectra before and after excitation with control light. (d) SHG enhancement factor γ as a function of input power/peak-intensity of the control (*P*_c_, *I*_c_) and seed (*P*_s_, *I*_s_) light. (e) Enhancement factor γ as a function of the control light power/intensity for *P*_s_ = 2 μW (data along the white dashed line in Figure 1d). (f) Polar plot of the circularly polarized SHG measured after passing a quarter-wave plate with and without the control light using σ- seed light. In (b)–(e), ℏω_c_ ≈ 3.1 eV. In (d)–(f), Δτ = 1.3 ps. In (b)–(f), ℏω_SHG_ ≈ 2.36 eV.

Figure 1a shows the schematic of our characterization setup, by which we study the SHG produced by seed light pulses as a function of delay time Δτ with respect to control light pulses in monolayer MoS_2_. All experiments are performed at ambient conditions (details in Methods and Supporting Information, SI). We present a typical SHG modulation result in Figure 1b. A readily available control light of photon energy ℏω_c_ ≈3.1 eV (λ_c_ ≈ 400 nm wavelength) above the C-exciton peak is chosen. The SHG signal at ℏω_SHG_ ≈ 2.36 eV (λ_SHG_ ≈525 nm) generated by the seed light at ℏω_s_ ≈ 1.18 eV (λ_s_ ≈ 1050 nm) is immediately enhanced by the control light with a single-exponential rising time constant (τ_0_ ≈ 600 fs, orange fitted line in Figure 1b). After Δτ ≈ 1.3 ps, the SHG intensity starts to decay, exhibiting two exponential time constants (τ_1_ ≈ 4.4 ps and τ_2_ ≈ 33 ps, red fitted curve in Figure 1b). The dynamics at different seed/control light powers (see Figure S3 in the SI) are similar to those shown in Figure 1b. The measured SHG spectra at Δτ = −1 and 1.3 ps are shown in Figure 1c, which reveals a strong SHG enhancement produced by the control light.

Figure 1d shows the enhancement factor γ at Δτ = 1.3 ps, where a maximum SHG signal is achieved, as a function of the control and seed light powers. We define the enhancement factor as γ = *P*_w_/*P*_wo_, where *P*_w_ and *P*_wo_ are the second harmonic (SH) powers measured with and without the control light, respectively. We find that γ is highly dependent on the incident light power. Figure 1e represents γ as a function of control light power when the average seed light power (peak intensity) is ∼2 μW (∼21.9 GW/cm^2^). We find that γ increases linearly with the control light power (*P*_c_) and is slightly saturated for *P*_c_ > 0.4 μW (corresponding to a light intensity of >3.43 GW/cm^2^, equivalent to an electron–hole (*e-h*) pair density of >5.7 × 10^14^ cm^–2^ when considering the measured absorption of ∼7.1% at ∼3.1 eV). In Figure 1e, we find that γ reaches a maximum value of 386 (with a corresponding enhancement of ∼19 times in second-order nonlinear optical susceptibility), which is ∼2 orders of magnitude larger than previously reported all-optical SHG enhancement results.^6,13−16,19−21,29^ We remark that the control light intensity is only ∼4.29 GW/cm^2^, that is, ∼5 times less than the seed light intensity of ∼21.9 GW/cm^2^. This is notable as the control light power is typically larger than the seed light power in the previously reported results.^30^ Similar enhancement phenomena (see Figure S3b in the SI) are observed when the control light energy is changed to ∼1.55 eV (λ_c_ = 800 nm). The maximum achievable γ at this lower photon energy of the control light is 75, that is, ∼5 times smaller than the results obtained with the 3.1 eV control light (Figure 1d). A higher incident control light power is required at 1.55 eV because it involves two-photon excitations. We have reliably repeated these results using different MoS_2_ flakes at ambient conditions with no observable change or damage. Larger γ is achieved with higher control power, as indicated in Figures 1d and S3b, although this results in gradual sample damage during the experiments.

Furthermore, we characterize the valley selection rule. By employing left-circularly polarized (σ−) seed light at ∼1.18 eV, the SH spectra filtered with σ– and σ+ polarizations show ∼96% helicity contrast (see Figure S10b in the SI), confirming the valley selection rule from the *D*_3*h*_ crystal symmetry (see the SI, Section 10).^21^ When switching the control light on and off, the polarization directions of SHG after passing a quarter-wave plate are almost the same with only ±2° variation (fitted parameters, Figure 1f), indicating that only σ+ polarized SHG is enhanced. This proves that symmetry remains conserved in the presence of control light excitation.

To explore the modulation mechanism, we measure the temporally and spectrally resolved SHG fractional power changes (Δ*P*_SHG_ = (*P*_w_ – *P*_wo_)/*P*_wo_) at different seed energies (ℏω_s_ from −0.92 to 1.44 eV) with a fixed control light energy of 1.55 eV. We note that the normalized time-resolved SHG dynamics with control light at 1.55 eV is similar to that observed at 3.1 eV (see Figure S4a in the SI). The former one allows us to precisely determine the zero-delay time by sum frequency generation in MoS_2_ (see Figure S5 in the SI).

**Figure 2:**
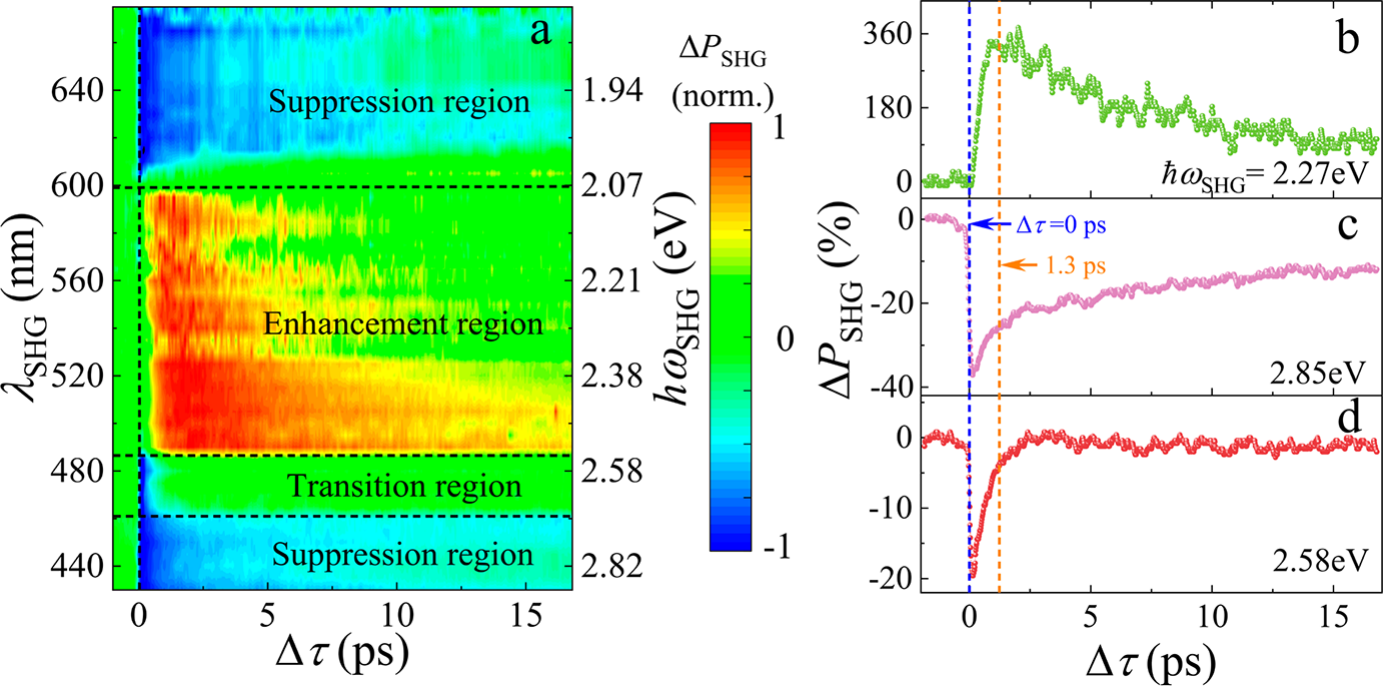
Broadband all-optical SHG modulation dynamics in MoS_2_. (a) Normalized SHG change Δ*P*_SHG_ as a function of time delay and SHG photon wavelength/energy. The vertical black dashed line indicates the position of Δτ = 0 ps. (b–d) Δ*P*_SHG_ response for ℏω_SHG_ ∼2.27, 2.85, and 2.58 eV, respectively. Blue and orange dashed lines mark Δτ = 0 and 1.3 ps. We use *I*_c_ ≈ 17.42 GW/cm^2^, ℏω_c_ ≈ 1.55 eV, and *I*_s_ ≈ 32.85 GW/cm^2^.

Figure 2a shows a broadband overview of the wavelength dependent SHG modulation dynamics. The SHG is enhanced (i.e., Δ*P*_SHG_ ≥ 0) by the control light when ℏω_SHG_ (λ_SHG_) lies in the ∼2.07–2.56 eV (∼598–485 nm) range. We denote this spectral range as the *enhancement region*. A representative result for ℏω_SHG_ ≈ 2.27 eV is plotted in Figure 2b, showing the dynamics similar to that in Figure 1b. When ℏω_SHG_ is in either the ∼2.64–2.88 eV (i.e., ∼470–430 nm) or the 1.84–2.07 eV (i.e., ∼675–598 nm) range, the SHG is reduced (i.e., Δ*P*_SHG_ ≤ 0) by the control light. We refer to this spectral range as the *suppression region* (Figure 2a). A representative result is shown in Figure 2c for ℏω_SHG_ ≈ 2.85 eV. In the suppression region, Δ*P*_SHG_ drops sharply in the presence of control light and reaches its minimum within a delay time Δτ ≈ 150 fs, faster than our experimental temporal resolution (see Figure S5a in the SI). Then, Δ*P*_SHG_ recovers with biexponential time constants τ_1_ ≈ 590 fs and τ_2_ ≈ 96 ps (see fitting details in Figure S4c in the SI). Within the range lying in between the above-mentioned enhancement and suppression regions in Figure 2a (i.e., ∼2.56–2.64 eV), our measurements reveal an extremely fast decay (Δτ ≈ 150 fs) followed by a fast recovery with a single-exponential time constant of ∼600 fs. An example of this behavior with ℏω_SHG_ ≈ 2.58 eV is offered in Figure 2d. We denote this spectral region as the *transition region*.

In our measurements (Figure 2), the time-resolved dynamics is almost independent of the seed and control light powers (see Figure S3 in the SI), whereas the relative SHG change (Δ*P*_SHG_) is linearly related with the control light power in all three regions using the 3.1 eV control light. Therefore, we can rule out an exciton–exciton interaction effect (e.g., exciton–exciton annihilation and Auger recombination), which would commonly exhibit a nonlinear excitation power dependence. We can thus attribute the SHG modulation effects (i.e., enhancement and suppression) to various excitonic transition processes (e.g., scattering, transition, and recombination) in monolayer MoS_2_.

**Figure 3:**
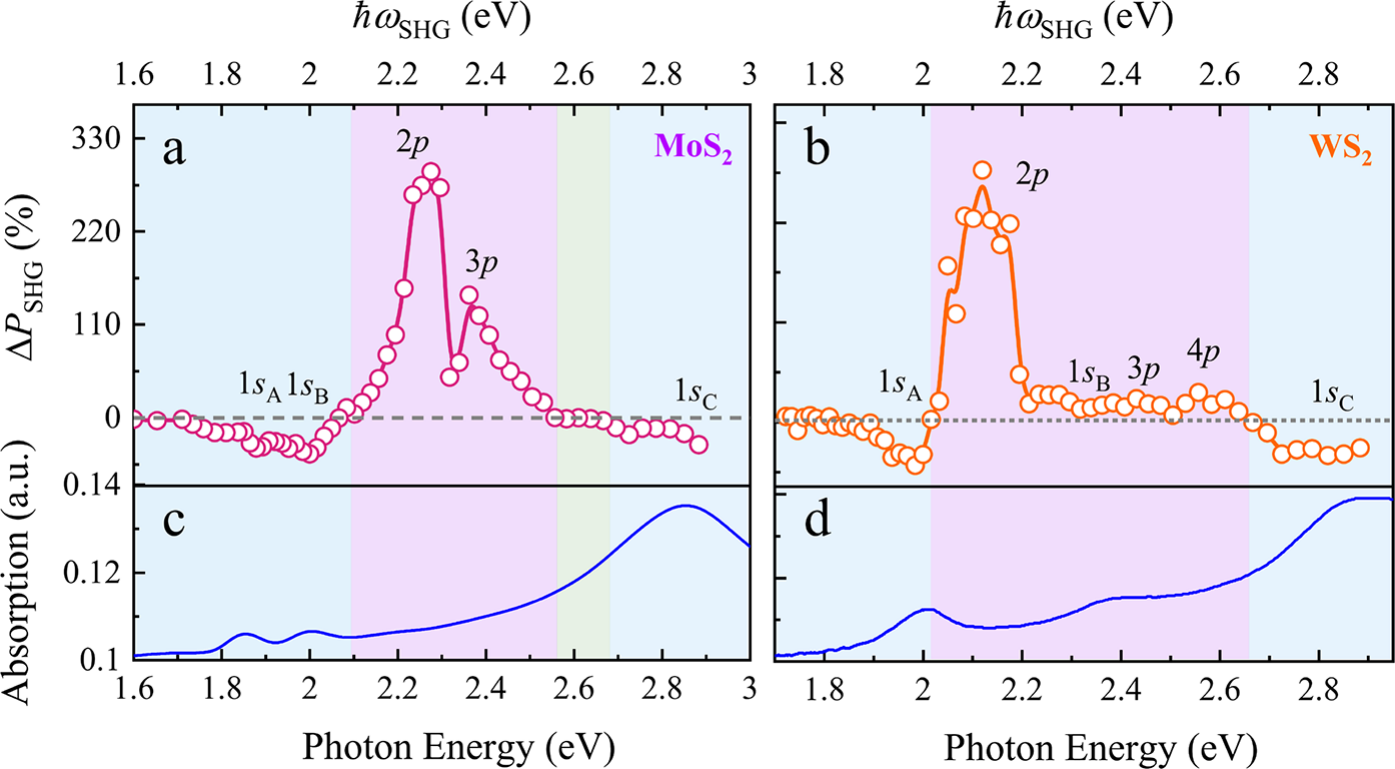
Maximum SHG modulation Δ*P*_SHG_ and optical absorption in monolayer MoS_2_ and WS_2_. (a) SHG change Δ*P*_SHG_ in monolayer MoS_2_ for Δτ = 1.3 ps, ℏω_c_ ≈ 1.55 eV, *I*_c_ ≈ 17.42 GW/cm^2^, and *I*_s_ ≈ 32.85 GW/cm^2^. (b) SHG change Δ*P*_SHG_ in monolayer WS_2_ for Δτ = 2.8 ps, ℏω_c_ ≈ 3.1 eV, *I*_c_ ≈ 2.57 GW/cm^2^, and *I*_s_ ≈ 32.85 GW/cm^2^. The gray dashed lines (zero value) and the solid curves connecting the dots are guides to the eye. (c, d) Linear optical absorption spectra of monolayer MoS_2_ and WS_2_, respectively. Different spectral regions are marked with background colors.

To gain further understanding, we plot Δ*P*_SHG_ as a function of the SHG photon energy (Figure 3a) for fixed seed and control light intensities with a delay Δτ ≈ 1.3 ps, where the maximum enhancement is achieved. We find that the minimum dip positions in the suppression region are well correlated with the energies of 1*s* bright exciton states (e.g., 1*s*_A_, 1*s*_B_, and 1*s*_C_, where the subscript denotes the exciton species) in the linear absorption spectrum of monolayer MoS_2_ (Figure 3c). We thus attribute the observed suppression of SHG to optical bleaching of bright excitons: the control light excites carriers from the ground state into quasi-continuum states with single-photon excitation processes at 3.1 eV (two-photon excitation at 1.55 eV), and the ground state becomes consequently depleted. This depletion inhibits the formation of bright excitons, blocking the typically observed bright excitonic enhancement effect of SHG and thus reducing the SHG signal.^11^ We provide a theoretical quantification of this effect below (see Methods). The bleaching process is typically fast (normally <100 fs),^31,32^ which fits well with the dynamics in the suppression region (Figure 2c). The subsequent biexponential recovery process in the suppression region can be correlated with different carrier relaxation processes, which gradually relax to the ground carrier states: an initial period of fast recovery with a characteristic time τ_1_ ≈ 590 fs can be attributed to carrier cooling dynamics and formation of bright excitons; a subsequent slow recovery with a time constant τ_2_ ≈ 96 ps can be attributed to carrier-phonon scattering and nonradiative carrier recombination. This biexponential recovery dynamics is similar to what has been previously reported in linear-absorption-based pump–probe measurements on bright excitons.^32,33^ We also note that we demonstrate electrical tunability of all-optical suppression of SHG at the 1*s*_A_ exciton of 1.89 eV (see Figure S9 in the SI), which holds great interest for on-chip electrically tunable all-optical nonlinear device applications. Our results demonstrate that electrical doping suppresses optical modulation, in analogy to electrically tunable SHG.^21^ This further confirms that the optically suppressed SHG effect is related to the bright excitons.

Additionally, we observe two strong enhancement peaks at ∼2.27 and 2.36 eV in Figure 3a, which are far away from the A and B excitons. We verify that these two enhancement peaks are not featured in either the linear interband absorption spectrum (Figure 3c) or the wavelength-dependent SHG spectrum (see Figure S8 in the SI). Furthermore, as shown in the time-resolved results of Figure 2a, the initial rise time of SHG modulation in the enhancement region (τ_0_ ≈ 600 fs, Figure 2b) is much longer than that in the suppression region (typically ∼150 fs, Figure 2c). This indicates a completely different carrier dynamics, which excludes various simultaneous or ultrafast nonlinear effects, including ultrafast optical bleaching and optical parametric interactions.^34^ At the same time, we do not observe any change in SHG modulation at 2.27 eV when applying electrical doping (i.e., for a back gate tuning voltage in the −100 to 100 V range). This indicates that electrical doping does not influence the SHG enhancement. In addition, by comparing the normalized SHG polarization dependence in monolayer MoS_2_ with and without the control light (see Figure S10a in the SI), we can exclude the possibility of a phase transition during the SHG enhancement process.

We also carry out SHG measurements in monolayer WS_2_ (see Figures S15 and S16 in the SI). We observe similar enhancement (with a measured γ reaching ∼70) and suppression effects in monolayer WS_2_, further corroborating the reported all-optical modulation as a general phenomenon in exciton-supporting TMDs. Figure 3b shows Δ*P*_SHG_ results at a delay time of 2.8 ps (where the maximum enhancement is obtained in Figure S16c in the SI). By comparing with the optical absorption spectral profile in Figure 3d, we assign the dip at ∼1.98 eV in the suppression region to an effect involving the bright 1*s*_A_ state, which also matches well with the PL measurements (see Figure S14 in the SI). In addition, the enhancement region in the Δ*P*_SHG_ spectrum ranging from ∼2.0 to 2.67 eV displays a strong peak at ∼2.11 eV and two small peaks at ∼2.43 and ∼2.58 eV, all of which are not visible in the linear interband absorption spectrum (Figure 3d).

**Figure 4:**
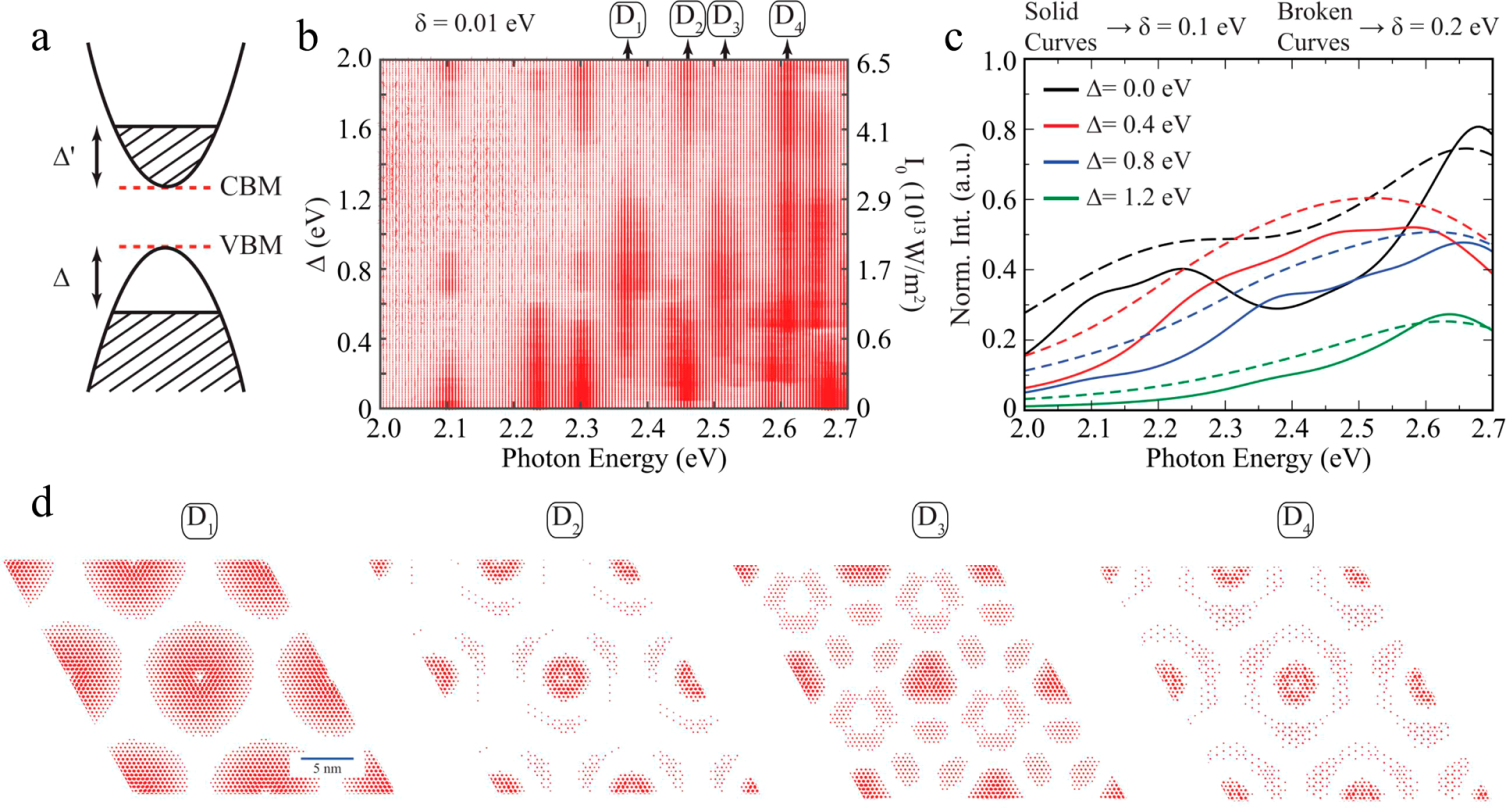
Theoretical interpretation of SHG modulation in monolayer MoS_2_. (a) Schematic representation of the electronic bands of monolayer MoS_2_ around the K point, showing an effective depletion region Δ produced upon optical pumping and a corresponding population of the conduction band up to an energy Δ′ adjusted to preserve the overall electron density. (b) Oscillator strength of excitonic states in the vicinity of the optically active region as a function of photon energy and Δ. (c) Spectrally resolved excitonic oscillator strength for Δ = 0, 0.4, 0.8, and 1.2 eV after introducing a photon energy broadening of 0.1 eV (solid curves) and 0.2 eV (broken curves). (d) Real-space wave functions of selected dark excitons, indicated by labels D_1_–D_4_ in Figure 4b, respectively.

To understand the observed optically driven SHG enhancement, we elaborate a theoretical interpretation of our experimental results in monolayer MoS_2_ based on first-principles calculations combined with a phenomenological treatment of optical pumping. We start by producing accurate calculations of the electronic band structure, as well as the exciton energies and wave functions (see Section S14 in the SI). We then introduce optical pumping through an effective depletion of electrons within an energy interval Δ at the top of the valence band, accompanied by the corresponding filling near the bottom of the conduction band (Figure 4a). The optical transition strengths associated with the excitons are then modified by this redistribution of band occupations, which we directly introduce in the electron–hole-pair (e−h) decomposition of their wave functions (see Methods). This allows us to produce a map of exciton transition strengths resolved in photon energy and band depletion energy Δ (Figure 4b). Spectral variations for selected values of Δ are shown in Figure 4c after introducing a spectral broadening to facilitate comparison to experiment. As the depletion energy increases, we find that the allowed excitations vary considerably: dark excitons with originally low oscillator strength increase their transition dipoles and dominate the optical spectrum, while bright excitons become weaker, in qualitative agreement with the experimental observations. We identify four dominant dark excitons in this process (D_1_–D_4_), whose real-space wave functions are potted in Figure 4d. We also note that there must be multiple excitations with low oscillator strength that may contribute to the SHG signal, but here we concentrate on the dominant excitations contributing to the observed effects. In addition, assuming that all of the energy absorbed by the material from the control light is invested in producing a depletion Δ (see Methods), we find that the required light intensities are a factor of ∼3 lower than those used in experiments (Figure 4b, right scale), which is reasonable in view of the fact that part of that energy can be lost through other dissipative processes (e.g., by spreading the energy among carriers away from the K point).

Supported by these theoretical calculations, we attribute the SHG enhancement to the modified exciton oscillation strength created by a redistribution of excited carriers. In the enhancement region, we propose that some of the carriers in the quasi-continuum of states excited by the control light scatter into low-energy bands and modify the *e-h* composition of the excited dark excitonic states, which acquire a substantial transition strength, thus playing a leading role in SHG (Figure 4b, c). We therefore attribute the rise time (e.g., τ_0_ = ∼600 fs in Figure 1b) to the remorphing of the *e-h* pair composition of dark excitons. The enhancement decreases due to decay of the excited carrier states with a relatively slow biexponential behavior (e.g., τ_1_ = ∼2.9 ps and τ_2_ = ∼325 ps at ∼2.27 eV, Figure 2b). We further attribute the fast decay to cooling dynamics of the excited dark excitons, while the slow recovery can be related to carrier-phonon scattering and nonradiative carrier recombination. We note that the decay time in the enhancement region is typically ∼10 times longer than the biexponential recovery components in the suppression region induced by bright excitons (Figure 2a, c). This fits well with the results of excited exciton dynamics observed in previous experiments.^22^ The leading role played by dark excitons can be further confirmed by comparing the enhancement peak positions with calculated dark exciton energies^35^ (see our comparison in Table S2 in the SI) and mid-infrared intraband absorption measurements (see details in Table S1 in the SI).^31^ We find that the two enhancement peaks at ∼2.27 and ∼2.36 eV are likely associated with the 2*p* and 3*p* excitonic states, respectively.

To explain the dynamics in the transition region, we plot time-resolved SHG modulation at different time delays in the ∼2.5–2.6 eV spectral range (see Figure S7g in the SI). The results confirm that the time-resolved SHG dynamics (Figure 2d) is governed by the contributions from the suppression and enhancement effects at different time scales: the fast suppression process (<150 fs) in the transition region is initially dominated by bright exciton suppression, similar to the suppression region; then, a relatively slow (∼1 ps) enhancement process takes over, similar to the initial response in the enhancement region.

The generality of the dark-exciton mechanism and the dynamics unveiled in this work is further supported by SHG experiments in monolayer WS_2_ (Figure 3b; see also Figures S14–S16 in the SI). We assign those peaks in the enhancement region as 2*p*, 3*p*, and 4*p* dark states by comparing with the energies of dark states from refs (28) and (36) (see Table S3 in the SI). This suggests that all-optical modulation of SHG is indeed applicable to other TMDs as well as their heterostructures. We also note that similar modulation effects are possible in other types of nonlinear optical processes, such as third harmonic generation, optical comb generation, and high harmonic generation, which deserve further investigation.

Although bright excitons (e.g., the 1*s* excitonic state) have been well studied already, dark excitons remain largely unexplored. This is because they are optically forbidden when relying on traditional interband absorption/emission-based pump–probe spectroscopy due to the optical selection rules.^28^ Here, thanks to our time-resolved SHG modulation method, we can access dark excitonic states and study their properties (e.g., population dynamics). The demonstrated method features two additional advantages for carrier dynamics exploration: First, its sensitivity is extremely high because the detection parameter of the modulation or change of the SHG signal can be extremely strong. For example, our modulation amplitude (i.e., the change in SHG intensity) is 4 orders of magnitude larger than the variation in the linear absorption (e.g., ∼0.2% at ∼2.27 eV in MoS_2_, as previously reported with traditional pump–probe spectroscopy^37^); secondly, the background noise is low because the detection signal is SHG, thus avoiding the strong probe signal background that is commonly encountered in traditional pump–probe spectroscopy.

## Conclusions

We have demonstrated giant all-optical modulation of SHG mediated by excitons in monolayer TMDs. The transient dynamics of excitonic dark and bright states in monolayer MoS_2_ has been determined to be the origin of the observed SHG modulation. Thanks to a redistribution of charge carriers produced by a control light beam, dark states acquire a substantial transition strength that contributes to enhance the SHG by a factor as large as 386 in our measurements. In addition, SHG is suppressed by applying electrical gating when the bright excitons are optically bleached. Our results on all-optical modulation of SHG provide a basis for exploiting the unique exciton-photon interactions in 2D materials, while they enable the development of emerging all-optical nonlinear optoelectronic applications.^38,39^ For example, the modulation amplitude, sign, and response time can be adjusted over a broad spectral range spanning a few electronvolts (see Figures 2a and S11). We have identified three observed regions with completely different SHG modulation responses that can potentially enable versatile photonic devices with different functionalities. In particular, an enhancement region that could be utilized for all-optically enhanced nonlinear processes with giant enhancement ratios by applying a relatively low control power. Also, a transition region, in which the large fractional SHG change |Δ*P*_SHG_| (up to 62%, equivalent to the modulation depth of an optical modulator, see Figure S6 in the SI) and the ultrafast fall (<150 fs) and rise (∼600 fs) response times could be used for ultrafast all-optical photonic devices, such as all-optical nonlinear modulators. Such a fast response time corresponds to a modulation speed of ∼1.4 THz, which is ∼14 times faster than that of state-of-the-art electro-optic modulators.^40^

## Methods

### Material Synthesis and Characterization

Monolayer MoS_2_ is grown on a SiO_2_/Si substrate by using the chemical vapor deposition method with an ∼10 mg sulfur (at 170 °C) and ∼0.5/15 mg NaCl/MoO_3_ mixture (at 750 °C) for 5 min in high purity argon.^41^ Optical characterization of MoS_2_, including Raman, photoluminescence, and reflection spectra, can be found in Figure S2 in the SI. A similar method is used to synthesize WS_2_, for which characterization is presented in Figure S14 in the SI.

### Experimental Methods

In the all-optical modulation experiment, the control and seed light pulses (2 kHz repetition rate) are generated by an optical parametric amplifier (Spectra-Physics, TOPAS) and divided into two parts using a dichroic mirror. The pulse duration of both control and seed pulses is ∼230 fs. The seed light goes through an optical delay line and is then combined with the control light by using another dichroic mirror (see Figure S1 in the SI). The combined beams are focused on the sample by a 40× objective of NA 0.75. The full-width-at-half-maximum beam diameters of the control light at 400 nm (800 nm) and the seed light are ∼2.5 and ∼2.2 μm, respectively. The generated SHG signal is then collected by a monochromator (Andor 328i). Different filters are used to remove the control and seed light before the monochromator. A photomultiplier tube (PMT, H7844 Hamamatsu) connected to a lock-in amplifier is used to detect and monitor the SHG signal. To calibrate the photon energy dependence, we extract the data after considering the whole system loss within the broad range of used photon energies and the optical reflectance/absorption of both MoS_2_ and the substrate.

### Theoretical Calculations

We model the pumping-dependent change in the exciton transition strengths from first principles assuming an effective depletion of the valence band produced by the control light. We obtain Kohn–Sham (KS) wave functions and eigenvalues by performing density-functional theory (DFT) calculations using the QUANTUM ESPRESSO code.^42^ We then use the Perdew–Burke–Ernzerhof (PBE) version of the generalized gradient approximation (GGA) for the exchange-correlation functional,^43^ combined with norm-conserving, fully relativistic pseudopotentials of the Pseudo-Dojo database.^44^ The plane-wave energy cutoff is set to 90 Ry for the ground-state calculations. We use the supercell method and include 45 atomic units of vacuum space between two periodic images of the semiconductor layer in order to minimize interactions between adjacent cells. Quasiparticle self-energy corrections to the KS eigenenergies are calculated within the many-body G_0_W_0_ approximation^45,46^ as implemented in the YAMBO code.^47^ The absorption spectrum and excitonic effects are obtained by solving the Bethe–Salpeter equation^48,49^ (BSE) on top of G_0_W_0_. The excitonic wave functions are described as |Φ^*S*^⟩ = Σ_*vck*_*A*_*vck*_^*S*^|*vck*⟩, where *v* and *c* denote valence and conduction band indices, *k* runs over wave vectors, *A*_*vck*_^*S*^ are expansion coefficients, and *S* is the exciton index. The excitation energies are determined by solving the BSE equations (*E*_*ck*_ – *E*_*vk*_)*A*_*vck*_^*S*^ + Σ_*v*′*c*′*k*′_⟨*vck*|*K*_eh_|*v*′*c*′*k*′⟩*A*_*v′c′k′*_^*S*^ = Ω^*S*^*A*_*vck*_^*S*^, where Ω*^S^* is the exciton eigenvalue, *E*_*vk*_ and *E*_*ck*_ denote the quasiparticle energies of valence and conduction electron band states, respectively, and *K*_eh_ is the electron–hole interaction kernel. We employ a wave vector grid consisting of 30 × 30 × 1 *k* points for both G_0_W_0_ and BSE calculations. The Coulomb cutoff technique is used at the edges of unit cells in the out-of-plane direction.^47^ We compute the self-energy and dynamical dielectric screening using 200 bands. The four highest valence bands and four lowest conduction bands are taken into account in the calculation of excitonic states.^50^

We simulate the optical transition strength in the presence of optical pumping by introducing an effective electron depletion near the top of the valence band, and correspondingly, an occupation near the bottom of the conduction band that preserves charge neutrality. More precisely, we calculate the pumping-dependent transition strength of exciton *S* using the expression *f*_*S*_ = |⟨*G*|***r⃗***|Φ̃*^S^*⟩|^2^/|⟨*G*|Φ̃*^S^*⟩|^2^, where |*G*⟩ denotes the ground state, whereas |Φ̃^*S*^⟩ = Σ_*vck*_*A*_*vck*_^*S*^*f*_*vk*_(1 – *f*_*ck*_)|*vck*⟩ is the exciton wave function obtained from its electron–hole-pair decomposition coefficients *A*_*vck*_^*S*^ and modified by electron redistribution according to the hole and electron occupations *f*_*vk*_ and *f*_*ck*_ that follow the band filling scheme shown in Figure 4a. For each given value of the valence depletion energy Δ, the conduction filling Δ′ is obtained by imposing charge neutrality through ∫_CBM_^CBM+Δ^′^^ d*E*_*ck*_ρ_*E*_*ck*__ = ∫_VBM−Δ_^VBM^ d*E*_*vk*_ρ_*E*_*vk*__, where VBM and CBM correspond to the valence band maximum and conduction band minimum, respectively, and ρ_*E*_ck__ and ρ_*E*_vk__ are the conduction and valence band densities of states, respectively. The depletion Δ is approximately related to the pumping light intensity *I*_0_ through the expression ∫_CBM_^CBM+Δ^′^^ d*E*_*ck*_*E*_*ck*_ρ_*E*_*ck*__ – ∫_VBM−Δ_^VBM^ d*E*_*vk*_*E*_*vk*_ρ_*E*_*vk*__ = *I*_0_*Aτ*_eff_, where *A* is the absorbance calculated at the pump energy ℏω_p_ = 3.1 eV and τ_eff_ is an effective electron–hole recombination time, which we set to an estimated value of 4 ps.^51^

## Supporting Information Available

Experimental setup; absorption, photoluminescence, and SHG spectra; experimental details of SHG modulation in MoS_2_ and WS_2_; additional calculations; supplementary discussions (PDF)
