# Supplementary material for: Giant All-Optical Modulation of Second-Harmonic Generation Mediated by Dark Excitons
Source: ACS Photonics. 2021 Jul 13;8(8):2320–8. doi: 10.1021/acsphotonics.1c00466 (PMC8377711; doi:10.1021/acsphotonics.1c00466)
Supplement: Supplementary file 1 — ph1c00466_si_001.pdf [file ph1c00466_si_001.pdf]

Supplementary Materials for

- **Giant all-optical modulation of second-harmonic generation mediated by dark excitons**

Yadong Wang[1,2,†], Susobhan Das[2,†], Fadil Iyikanat[3,†], Yunyun Dai[2], Shisheng Li[4], Xiangdong Guo[5], Xiaoxia Yang[5], Jinluo Cheng[6], Xuerong Hu[2,7], Masood Ghotbi[8], Fangwei Ye[9], Harri Lipsanen[2], Shiwei Wu[10], Tawfique Hasan[11], Xuetao Gan[1], Kaihui Liu[12], Dong Sun[13], Qing Dai[5], F. Javier García de Abajo[3,14,*], Jianlin Zhao[1,*] and Zhipei Sun[2,15,*]

[1]MOE Key Laboratory of Material Physics and Chemistry under Extraordinary Conditions, and Shaanxi Key Laboratory of Optical Information Technology, School of Physical Science and Technology, Northwestern Polytechnical University, Xi'an 710129, China

[2]Department of Electronics and Nanoengineering, Aalto University, Espoo 02150, Finland

[3]ICFO-Institut de Ciencies Fotoniques, The Barcelona Institute of Science and Technology, 08860 Castelldefels (Barcelona), Spain

[4]International Center for Young Scientists, National Institute for Materials Science, Tsukuba, Japan

[5]Division of Nanophotonics, CAS Center for Excellence in Nanoscience, National Center for Nanoscience and Technology, Beijing 100190, China

[6]Changchun Institute of Optics, Fine Mechanics and Physics, Chinese Academy of Sciences, Changchun, Jilin, China.

[7]International Cooperation Base of Photoelectric Technology and Functional Materials, and Institute of Photonics and Photon-Technology, Northwest University, Xi'an 710069, China

[8]Department of Physics, University of Kurdistan, Pasdaran St., Sanandaj, Iran

[9]School of Physics and Astronomy, Shanghai Jiao Tong University, Shanghai 200240, China

[10]State Key Laboratory of Surface Physics, Key Laboratory of Micro and Nano Photonic Structures (MOE), and Department of Physics, Fudan University, Shanghai 200433, China

[11]Cambridge Graphene Centre, University of Cambridge, Cambridge CB3 0FA, UK.

[12]State Key Laboratory for Mesoscopic Physics and School of Physics, Peking University, Beijing 100871, China

[13]International Center for Quantum Materials, School of Physics, Peking University, Beijing 100871, China

[14]ICREA-Institució Catalana de Recerca i Estudis Avançats, Passeig Lluís Companys 23, 08010 Barcelona, Spain

[15]QTF Centre of Excellence, Department of Applied Physics, Aalto University, Espoo, Finland

[*]Emails: zhipei.sun@aalto.fi, jlzhao@nwpu.edu.cn, javier.garciadeabajo@nanophotonics.es

[†]Those authors contributed equally to this paper.

1. Experimental setup

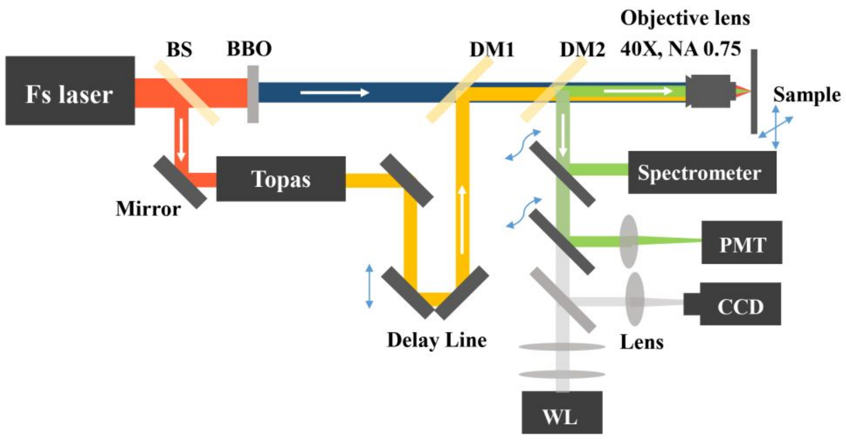

Fig. S1. Scheme of the experimental setup. The femtosecond laser source is Solstice Ace from Spectra-Physics. The TOPAS system is used for frequency conversion to produce the seed light with a wavelength range of 820-1600 nm. The control light wavelengths are either 400 nm or 800 nm. The pulse duration is ~230 fs and the repetition rate is 2 KHz. BBO: Beta barium borate; WL: white light for optical imaging of the sample; DM: dichroic mirror; BS: beam splitter; PMT: photomultiplier.

2. Monolayer MoS_2 sample characterization

The MoS_2 sample is prepared by chemical vapor deposition (CVD). Figure S2a shows a typical triangular flake deposited on a SiO_2/Si substrate. The Raman spectrum in Fig. S2b shows peaks at ~383 and 402 cm^{-1}, with a difference of 19 cm^{-1} identifying monolayer MoS_2. As shown in Fig. S2c, photoluminescence (PL) mapping shows that the MoS_2 layer is grown well and uniformly. Furthermore, the measured PL spectrum reveals two strong peaks at 620 nm and 670 nm (fitted by Lorentzians), corresponding to B and A excitons, respectively, as shown in Fig.

S2d. Photoluminescence and Raman spectra are measured under excitation with a continuous-wave laser of 532 nm wavelength and 0.8 μW incident power focused with a NA of 0.75.

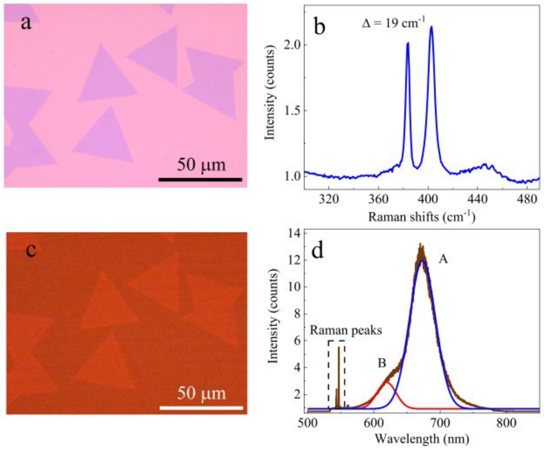

Fig. S2. CVD MoS_2 optical characterization. (a) Optical image. (b) Raman spectrum. (c) Photoluminescence mapping at the same position as in (a). (d) Photoluminescence spectrum.

3. Control and seed light power dependence of the SHG enhancement factor and time-resolved dynamics

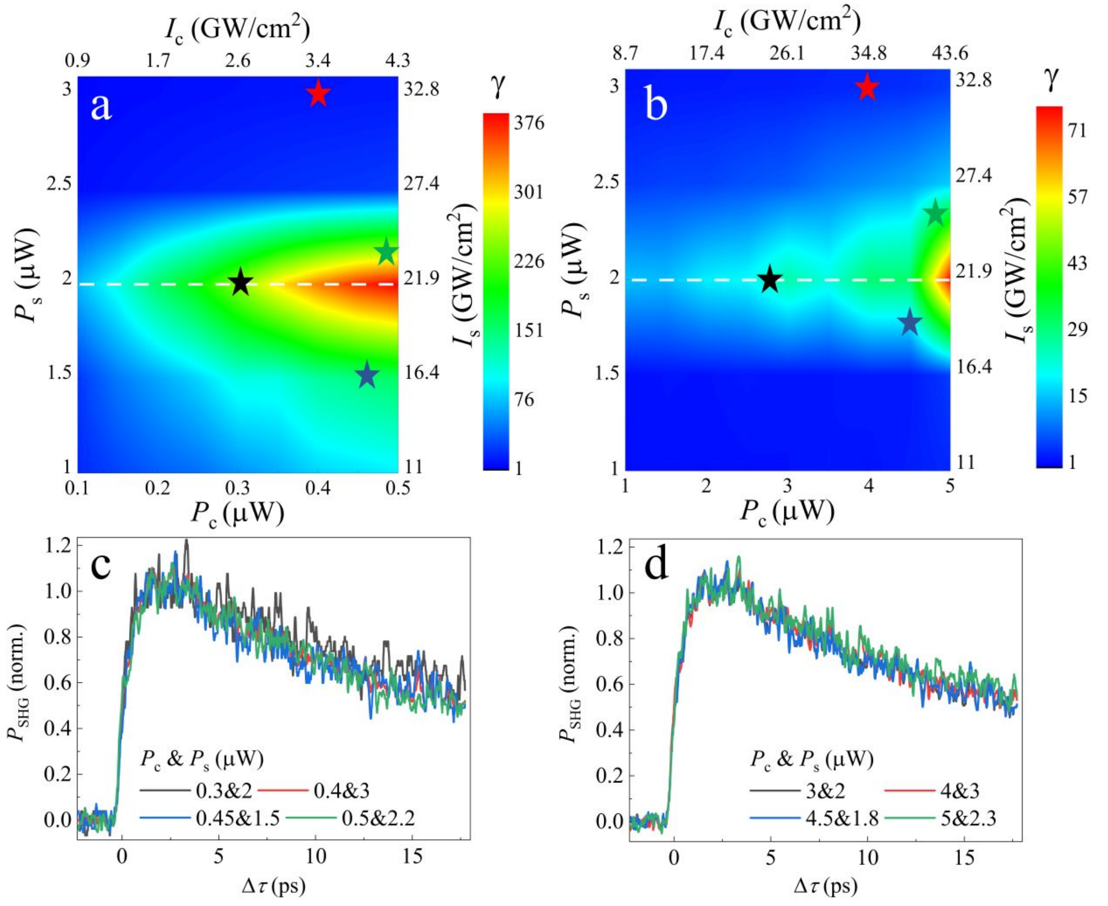

Fig. S3. (a,b) Power dependence of the enhancement factor γ for a control-seed delay $\Delta\tau$=1.3 ps with control light of wavelength λ_c =400 nm (a) and λ_c =800 nm (b). (c,d) Normalized P_{SHG} dynamics with different control light powers P_c and seed light powers P_s for λ_c = 400 nm (c) and λ_c = 800 nm (d). The dynamics of P_{SHG} is shown in (c) and (d), respectively The positions of stars in (a) and (b) correspond to different control and seed powers. The colors of stars and dynamics curves are coordinated.

4.Time-resolved ΔP_{SHG} dynamics in different regimes

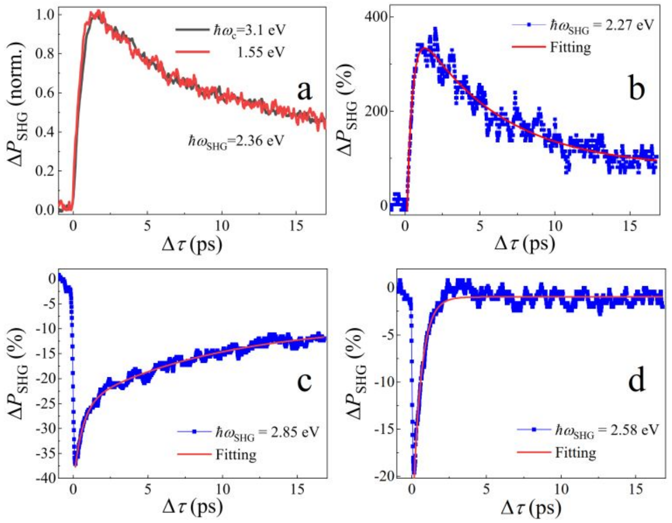

Fig. S4. (a) Time-resolved ΔP_{SHG} dynamics for two different control light photon energies (black: 3.1 eV; red: 1.55 eV) with seed light energy of 1.18 eV, which show a very similar transient response of ΔP_{SHG}. (b)-(d) Time-resolved ΔP_{SHG} dynamics with exponential fittings for three representative seed light energies in three different regimes: enhancement, suppression, and transition, respectively; the control light photon energy is fixed at 1.55 eV.

5. Photo-generation and recombination of charge carriers in transient ΔP_{SHG}

Figure S5 shows detailed measurements of carrier dynamics through ΔP_{SHG}. Figures S5a and S5c show the transient dynamics of ΔP_{SHG} when the SHG energies are 2.85 eV and 2.36 eV, respectively. The yellow curve is the sum frequency generation (SFG) measured under the same conditions to characterize the pulse duration. It shows that the drop time in Fig. S5a is much shorter than the control-seed light cross-correlation curve, while the decay time shows slower response, indicating the presence of different dynamical carrier processes. In contrast, in Fig. S5c ΔP_{SHG} at 2.36 eV photon energy initially rises until the delay time reaches $\Delta\tau = 1.3$ ps, and then

it starts to decay. We note that the rise time is longer than the pulse duration. Figures S5b and S5d show the relationship between the control light power and the SHG signal for $\Delta\tau = 0.15$ ps (Fig. S5a) and 1.3 ps (Fig. S5c).

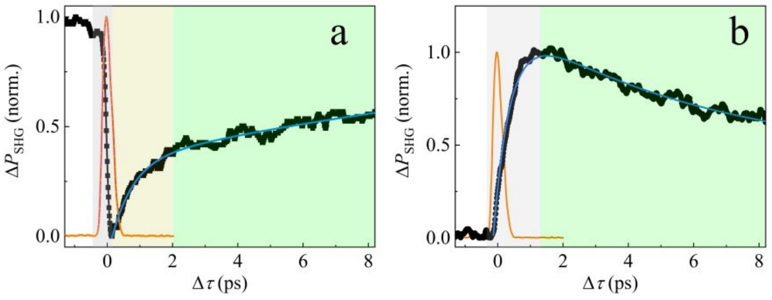

Fig. S5. Transient ΔP_{SHG} response for $\hbar\omega_{SHG} = 2.85$ eV (a) and 2.36 eV (b) with the corresponding seed light photon energies at ~1.425 eV and 1.18 eV. The blue curves are exponential fittings of the decay processes. The orange curves indicate the signal of sum frequency generation (lying between the control light at 1.55 eV and the seed light), which is measured simultaneously during the all-optical modulation experiment. The peak positions of the sum frequency generation (orange curves) provide a good reference for the zero-time delay between the control and seed pulses.

6. Modulation of ΔP_{SHG} with control light power in the transition region

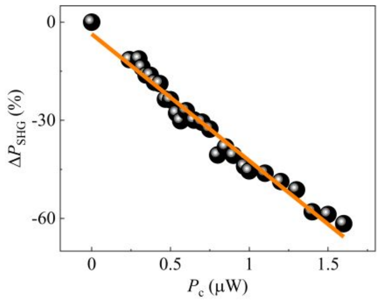

Fig. S6. Linear power dependence for $\hbar\omega_{SHG}$ = 2.58 eV and 2 μW average incident power of the seed light. The control light photon energy is $\hbar\omega_c$ = 3.1 eV.

7. Time-resolved ΔP_{SHG} spectra

In Figure S7, we plot results from SHG modulation spectroscopy at different time delays in the spectral range from ~2.16 to 2.88 eV. The spectra show very clear dynamics when Δτ varies from -1 to 10 ps (Fig. S7a-f). We observe that the enhancement peak positions remain unchanged when introducing control light, indicating that bandgap renormalization does not affect the sign of the change in SHG modulation. Furthermore, detailed spectra near the ΔP_{SHG}=0 condition are measured and shown in Fig. S7g, which shows blue shifts due to the evolution from the suppression region to the enhancement region.

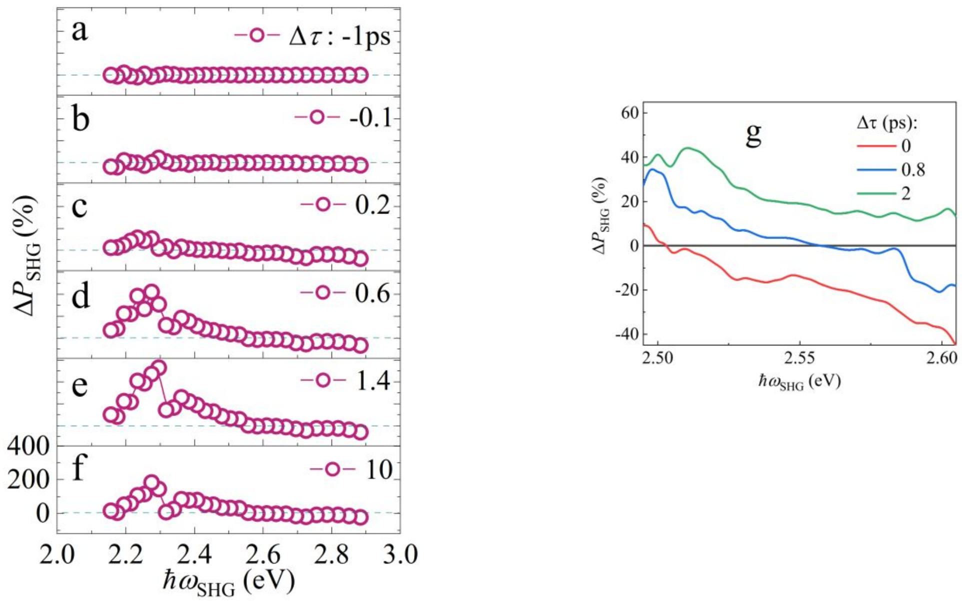

Fig. S7. (a)-(f) ΔP_{SHG} spectra at different delays $\Delta\tau$. (g) Blue shift of ΔP_{SHG} spectra at different time delays when $\hbar\omega_{\mathrm{SHG}}$ lies in the transition region.

8. Comparison between SHG and ΔP_{SHG} in MoS_2

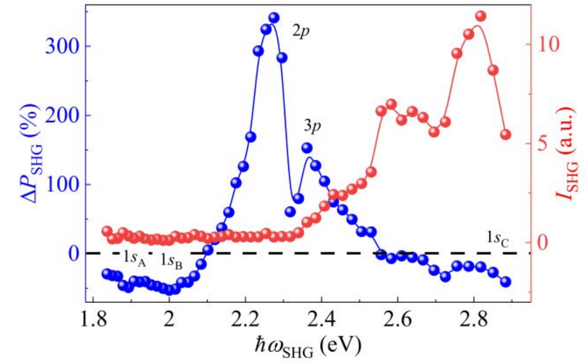

Fig. S8. Comparison between ΔP_{SHG} at $\Delta\tau = 1.1$ ps and the SHG responses of monolayer MoS_2. We attribute the larger SHG responses observed at higher photon energies (e.g., at 2.8 eV) to the C-exciton resonance.

9. MoS_2 field-effect transistor and electrically tunable all-optical modulation of SHG

It has been previously reported that exciton resonances and electrical doping can change the SHG response in monolayer TMDs.[1] We therefore investigate the possibility of electrical tunability of our observation of all optical SHG modulation. To this end, we have fabricated a MoS_2 field-effect transistor (Fig. S9a) for the experiments. The two electrodes are first patterned using electron-beam lithography (Vistec 5000+ES, Germany) and then covered with Ti (10 nm)/Au (60 nm) using an electron beam evaporation (OHMIKER-50B, Taiwan). The electrical performance of this device is shown in Fig. S9b. In particular, ΔP_{SHG} at the $1s_A$ exciton (1.89 eV energy) with different gate voltages (V_g) is shown in Fig. S9c. Figure S9d shows that the maximum $|\Delta P_{SHG}|$ varies from 43% to 25% at constant delay $\Delta\tau \approx 150$ fs when V_g increases from 0 to 100 V. These results indicate that electrical doping induces suppression of optical modulation, similar to the electrically tunable SHG behavior.[1] Electrical doping changes the Fermi level, which in turn influences the carrier occupation and thus the population of excitons.[1] Therefore, changing the doping level can eventually tune optical modulation of SHG in the material under investigation.

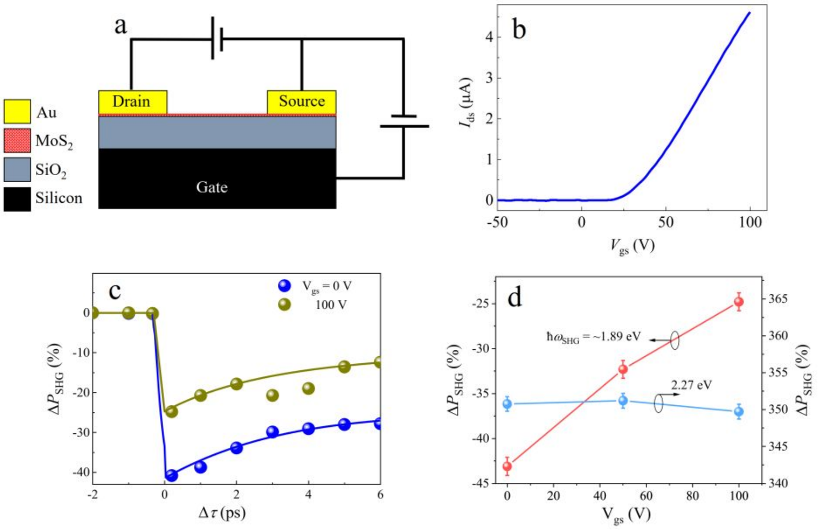

Fig. S9. (a) Schematic illustration of a MoS_2 field-effect transistor. (b) Source-drain current (I_{ds}) versus gate voltage (V_{gs}) when the drain voltage is fixed to $V_{ds} = 1$ V. (c) Optical modulation at different gate voltages for $\hbar\omega_{SHG} \approx 1.89$ eV. The applied seed and control light intensities are ~54.75 GW/cm^2 and ~17.42 GW/cm^2, respectively. (d) ΔP_{SHG} as a function of gate voltage when $\Delta\tau = \sim150$ fs.

10. Preservation of MoS_2 symmetry with control light

We perform circularly-polarized SHG measurements in which circularly-polarized seed light is generated by combining a linear polarizer and a quarter-wave plate, focused on the monolayer MoS_2 with a sapphire substrate. The generated SHG is collimated with an objective lens and converted into linear polarization after going through another quarter-wave plate. Finally, a linear polarizer is employed to measure the polarization angle of the converted SHG signal.[2]

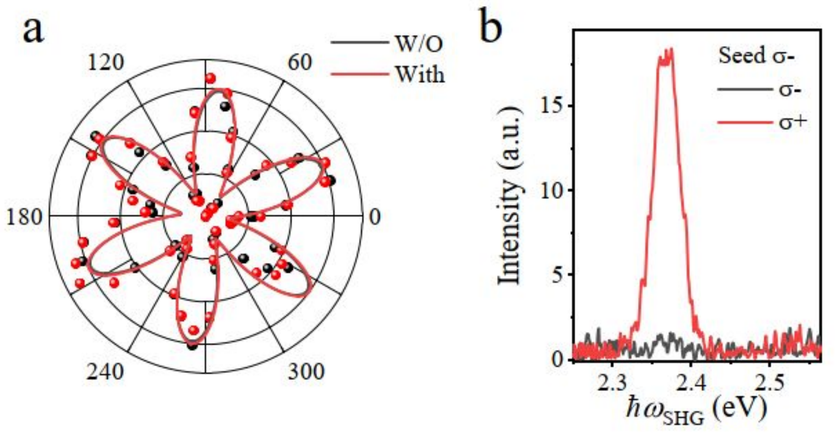

Fig. S10. (a) Polar plots of the normalized SHG intensity as a function of sample angle with (red curve) and without (black curve) pump excitation when the seed light is linearly polarized. (b) Circular polarization-resolved SHG spectra of the $3p$ excitonic state with excitation by a left-circularly-polarized seed laser (σ-).

11. Comparison of measured excitonic states with previous experimental results

TABLE S1. Comparison of exciton energies in monolayer MoS_2.

Exciton	Energy from Ref. 3 (eV)	Energy from ΔP_{SHG} (eV)※	Energy from the linear reflection (eV)
$1s_A$	1.86	1.89	1.9
$2p_A$	-	-	-
$3p_A$	2.13	-	-
$1s_B$	2.01	2.0	2.05
$2p_B$	2.22	2.27	-
$3p_B$	2.37	2.36	-
$1s_C$	-	2.9	2.93

※This work

12. Sign change in the enhancement and suppression regions for various SHG wavelengths

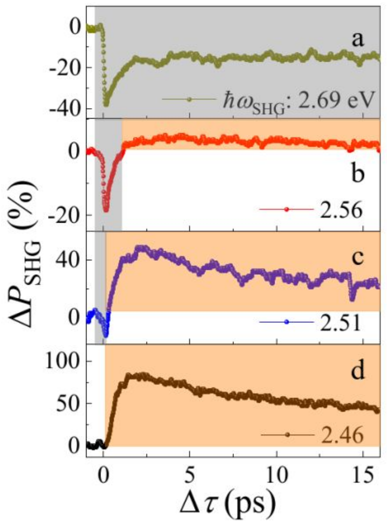

Fig. S11. (a)-(d) ΔP_{SHG} responses when varying $\hbar\omega_{SHG}$ in the 2.46-2.69 eV range. Negative and positive signs are indicated by grey and orange regions.

13. Theoretical calculation of the change in second-order nonlinear susceptibility

The modulation of the SHG signal before and after the arrival of a control light pulse can be explained as the change in the second-order nonlinear susceptibility of the system $\chi^{(2)}$. The power of the SHG signal generated from the seed light in the absence of control light (p_0) or in the presence of control light (p_c) can be expressed as

$$p_0 \propto \left(\chi_0^{(2)}\right)^2$$

$$p_c \propto \left(\chi_c^{(2)}\right)^2$$

where $\chi_c^{(2)}$ and $\chi_0^{(2)}$ are the corresponding second-order nonlinear susceptibilities of monolayer MoS_2 at the seed wavelength with and without control light, respectively.

From here, the relative change in second-order nonlinear susceptibility can be expressed as

$$\frac{\Delta\chi^{(2)}}{\chi_0^{(2)}} = \frac{\chi_c^{(2)} - \chi_0^{(2)}}{\chi_0^{(2)}} = \sqrt{\frac{p_c}{p_0}} - 1$$

In the enhancement region, we find a maximum relative change in second.order nonlinear susceptibility of ~19.

14. Supplementary theoretical elements in the analysis of SHG modulation

We use the GW-BSE method to calculate the absorption spectrum of monolayer MoS_2. This method yields reliable predictions for the excited-state properties of ultra-thin transition metal dichalcogenides.[4, 5] In Fig. S12a, we represent the electronic band structure calculated within the GGA and G_0W_0 approximations for monolayer MoS_2, showing that inclusion of quasiparticle energy corrections to the Kohn-Sham eigenvalues (i.e., when moving from GGA to G_0W_0) leads to a direct band gap of 2.77 eV at the K point.

We obtain the absorbance spectrum of monolayer MoS_2 from the imaginary part of the dielectric function, as shown in Fig. S12b, which reveals several excitonic features located below the band gap of the material with high oscillator strengths. A comparison of the spectral peak positions of the experimentally observed and theoretically predicted bright excitonic states is presented in Table S2. The 0.25 eV redshift of the calculated peak positions relative to experiment can be attributed to the error margin of the GW-BSE approach.[6] In addition, we show the variation of band gap and binding energy of the two lowest-energy excitons ($1s_A$ and $1s_B$) with respect to the size of the *k*-point mesh and the number of bands in Fig. S13.

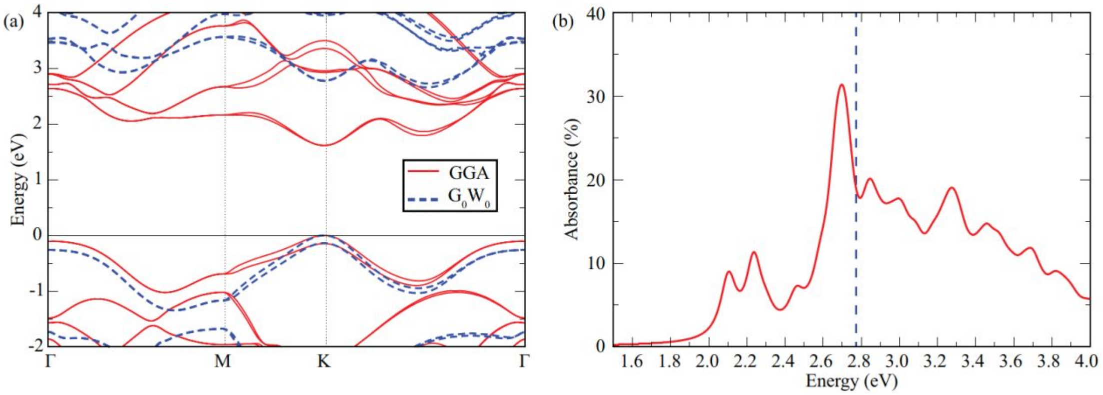

Fig. S12. (a) Electronic bands of monolayer MoS_2 calculated in the GGA and G_0W_0 approximations. (b) Absorbance of monolayer MoS_2 computed within the G_0W_0−BSE approximation.

TABLE S2. Comparison of experimental and theoretical exciton energies in monolayer MoS_2.

	**1s_A**	**1s_B**	**2p_A**	**2*s***	**3p_A**	**2p_B**	**3*s***	**3p_B**
**Bright(B)/Dark(D)**	B	B	D_1	B	D_2	D_3	B	D_4
**Experiment (eV)**	1.85	2.00	-	2.19	-	2.27	2.36	2.37
**Calculation (eV)**	2.10	2.24	2.37	2.44	2.46	2.52	2.59	2.59

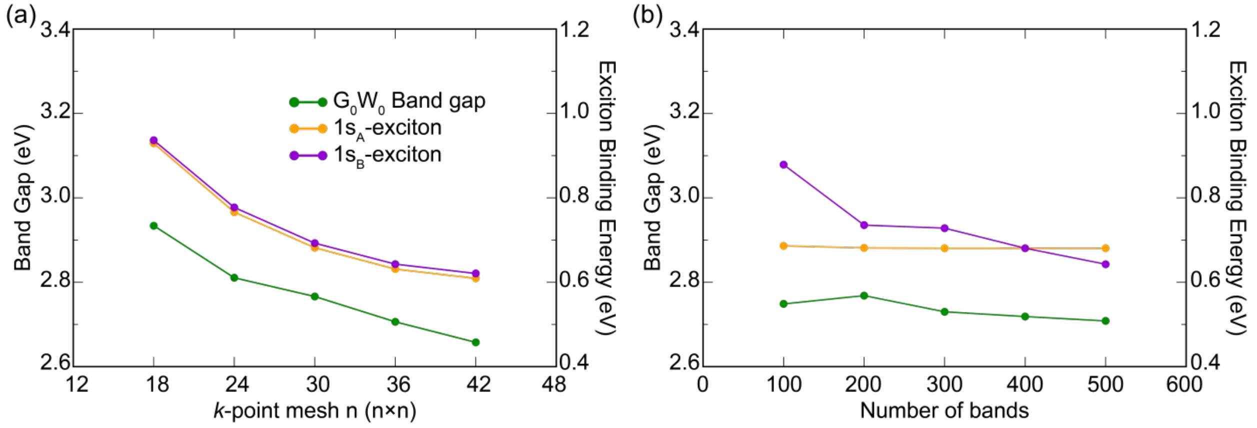

Fig. S13. Convergence of the direct band gap at the K-point and the binding energies of $1s_A$ and $1s_B$ excitons with respect to (a) the size of the k-point mesh and (b) the number of bands. In each panel, left and right scales show the band gap and exciton binding energies, respectively.

15. SHG modulation with monolayer WS_2

A typical WS_2 sample characterization is shown in Fig. S14.

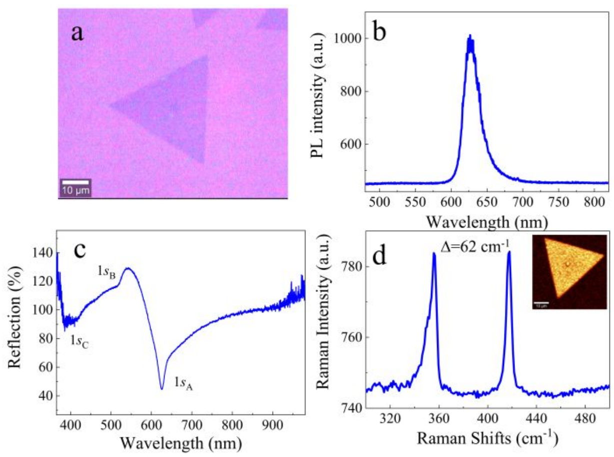

Fig. S14. CVD WS_2 sample characterization. (a) Optical image. (b) Photoluminescence spectrum. (c) Optical reflectance. (d) Raman spectrum with an excitation wavelength of 488 nm. Inset: Raman map of the 336 cm^{-1} shifted peak.

Our measurements of SHG modulation in WS_2 are carried out with the same system employed for MoS_2 using control light of 400 nm wavelength (3.1-eV photon energy). Figure S15a shows the dependence of SHG enhancement γ on the power of control and seed light (1170 nm, 1.05 eV). Clearly, the SHG enhancement factor γ increases with increasing control light power, whereas it decreases with increasing seed light power. Figure S15b shows γ as a function of control light power when the seed light power is 1 μW, from which we conclude that γ can be as high as ~76 when the control light power is 0.3 μW.

The normalized ΔP_{SHG} is shown in Fig. S16 as a function of time delay for different seed light wavelengths. The incident powers of the seed light and control light are 3 μW and 0.3 μW, respectively. As shown in Fig. S16a, the time-resolved ΔP_{SHG} dynamics with various SHG energies clearly reveals the presence of suppression, transition, and enhancement regions. The results of SHG modulation presented in Fig. S16b-e show very similar dynamics when compared to those for MoS_2 at different regions.

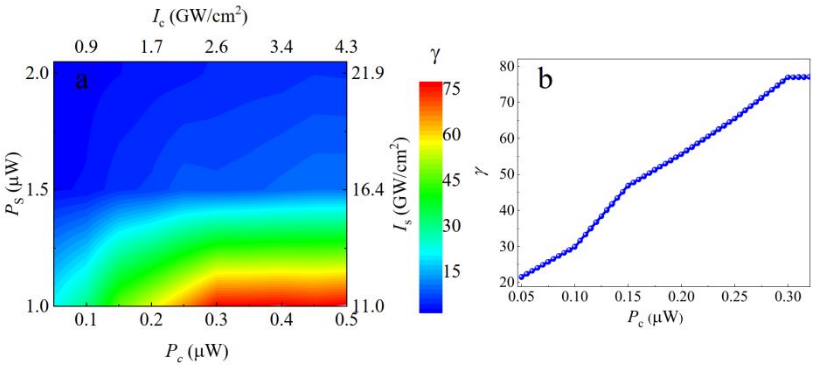

Fig. S15. (a) SHG enhancement factor γ as a function of input power/peak-intensity of the control (P_c, I_c) and seed (P_s, I_s) light in WS_2. (b) γ as a function of control light power for a seed light power of 1 μW.

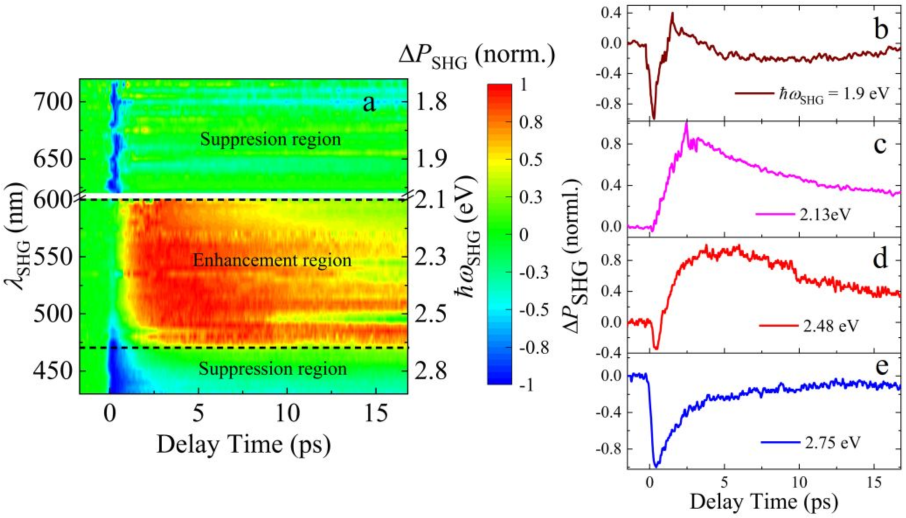

Fig. S16. All-optical modulation dynamics in monolayer WS_2. (a) Normalized SHG power change ΔP_{SHG} as a function of time delay and SHG photon wavelength/energy. The breaking region signals the overlap between difference frequency generation and SHG. (b)-(e) ΔP_{SHG} dynamics for $\hbar\omega_{SHG}$ = 1.9, 2.13, 2.48, and 2.75 eV, respectively.

TABLE S3. Comparison of exciton energies in monolayer WS_2.

	**$1s_A$**	**$2p$**	**$1s_B$**	**$3p$**	**$4p$**	**T**
Ref. S5	2.04	2.28	2.45	2.48		10k
Ref. S7	2.12	*	2.50	*	*	10k
Ref. S7	2.02	*	2.40	2.45	2.58	300k
Linear	1.98	*	2.39	*	*	300k
ΔP_{SHG}	1.98	2.11	*	2.43	2.58	300k

Supplementary References

(S1) Seyler, K. L.; Schaibley, J. R.; Gong, P.; Rivera, P.; Jones, A. M.; Wu, S.; Yan, J.; Mandrus, D. G.; Yao, W.; Xu, X., Electrical control of second-harmonic generation in a WSe_2 monolayer transistor. *Nat. Nanotechnol.* **2015**, *10* (5), 407-11.

(S2) Zhang, D.; Zeng, Z.; Tong, Q.; Jiang, Y.; Chen, S.; Zheng, B.; Qu, J.; Li, F.; Zheng, W.; Jiang, F.; Zhao, H.; Huang, L.; Braun, K.; Meixner, A. J.; Wang, X.; Pan, A., Near-Unity Polarization of Valley-Dependent Second-Harmonic Generation in Stacked TMDC Layers and Heterostructures at Room Temperature. *Adv. Mater.* **2020**, *32* (29), e1908061.

(S3) Cha, S.; Sung, J. H.; Sim, S.; Park, J.; Heo, H.; Jo, M. H.; Choi, H., 1s-intraexcitonic dynamics in monolayer MoS_2 probed by ultrafast mid-infrared spectroscopy. *Nat. Commun.* **2016**, *7*, 10768.

(S4) Qiu, D. Y.; da Jornada, F. H.; Louie, S. G., Optical spectrum of MoS_2: many-body effects and diversity of exciton states. *Phys. Rev. Lett.* **2013**, *111* (21), 216805.

(S5) Ye, Z.; Cao, T.; O'Brien, K.; Zhu, H.; Yin, X.; Wang, Y.; Louie, S. G.; Zhang, X., Probing excitonic dark states in single-layer tungsten disulphide. *Nature* **2014**, *513*, 214.

(S6) Molina-Sanchez, A.; Sangalli, D.; Hummer, K.; Marini, A.; Wirtz, L., Effect of spin-orbit interaction on the optical spectra of single-layer, double-layer, and bulk MoS_2. *Phys. Rev. B* **2013**, *88* (4), 045412.

(S7) Zhu, B.; Chen, X.; Cui, X., Exciton binding energy of monolayer WS_2. *Sci Rep-Uk* **2015**, *5*, 9218.
